# GTF2IRD1 overexpression promotes tumor progression and correlates with less CD8^+^ T cells infiltration in pancreatic cancer

**DOI:** 10.1042/BSR20202150

**Published:** 2020-09-28

**Authors:** Hongkai Zhuang, Chuanzhao Zhang, Baohua Hou

**Affiliations:** Department of General Surgery, Guangdong Provincial People’s Hospital, Guangdong Academy of Medical Sciences, Guangzhou 510080, China

**Keywords:** CD8+ T cells, GTF2IRD1, pancreatic cancer, T cell receptor signaling pathway, TGF-β

## Abstract

**Background:** General Transcription Factor II-I Repeat Domain-Containing Protein 1 (GTF2IRD1) is a member of the GTF21 gene family, which encodes a set of multifunctional transcription factors. However, the potential function of GTF2IRD1 in pancreatic cancer (PC) still remains unknown. Study on GTF2IRD1 might provide a new insight into the carcinogenesis and therapeutics of PC.

**Methods:** In the current study, the clinical significance and potential biological of GTF2IRD1 were evaluated by bioinformatics analysis. The oncogenic role of GTF2IRD1 in PC was also determined using *in vitro* studies. Possible associations between GTF2IRD1 expression and tumor immunity were analyzed using ESTIMATE algorithm and single-sample Gene Set Enrichment Analysis (ssGSEA).

**Results:** GTF2IRD1 expression was significantly up-regulated in tumor tissues, and positively associated with higher histologic grade, higher American Joint Committee on Cancer (AJCC) stage, and worse prognosis. Function enrichment analysis demonstrated that GTF2IRD1 may be involved in pancreatic adenocarcinoma pathway, TGF-β signaling pathway, and tumor-infiltrating lymphocyte (TIL) related biological functions, such as T-cell receptor signaling pathway, leukocyte transendothelial migration, resistin as a regulator of inflammation, and regulation of leukocyte-mediated cytotoxicity. Knockdown of GTF2IRD1 expression inhibited cancer cell proliferation, colony formation, and invasion *in vitro*. ESTIMATE algorithm and ssGSEA demonstrated that GTF2IRD1 expression negatively correlated with the infiltration and anti-tumor activity of TILs, especially for CD8^+^ T cells.

**Conclusion:** The study demonstrates that GTF2IRD1 overexpression promotes tumor progression and correlates with less CD8^+^ T cells infiltration in PC.

## Introduction

As one of the most aggressive malignancies, pancreatic cancer (PC) causes 4.5% of all cancer-related deaths worldwide in 2015 [1]. PC is widely recognized as a ‘cold’ tumor due to its notable immunosuppression [2]; thus, enhancing or recruiting anti-tumor immune cells into the tumor microenvironment (TME) is a promising therapeutic strategy for PC [3]. In recent years, immune checkpoint inhibitors (ICIs) have been effectively used for several solid tumors, such as melanoma, non-small cell lung cancer, and hepatocellular carcinoma [4–6]. However, these drugs fail to be effective treatments for patients with advanced PC due to the lack of CD8^+^ T cells in the TME of PC [7]. Therefore, exploring the molecular mechanisms underlying PC progression and immune suppression is critical for developing more effective immunotherapies to improve survival rates.

General Transcription Factor II-I Repeat Domain-Containing Protein 1 (GTF2IRD1) located at 7q11.23, is a member of the GTF21 gene family, which encodes a set of multifunctional transcription factors [8]. Early studies on GTF2IRD1 mainly focused on its association with Williams–Beuren syndrome, which is a genetic disorder associated with multiple systemic abnormalities, including hypertension, diabetes, osteoporosis, and anxiety [9,10]. Previous oncology studies about GTF2IRD1 have been conducted in only three cancers, including colorectal cancer, breast cancer, and retinoblastoma [8,11]. Nevertheless, the precise function and mechanism of GTF2IRD1 in PC has not been studied to date. Besides, the potential association between GTF2IRD1 expression and immune infiltration in PC still remain to be elucidated.

In the present study, for the first time, we comprehensively analyzed the expression of GTF2IRD1 and its correlation with prognosis in PC. Then, we also analyzed the potential underlying biological role of GTF2IRD1 in PC using functional enrichment analysis in ConsensuspathDB (http://cpdb.molgen.mpg.de/). And the oncogenic role of GTF2IRD1 in PC was determined using *in vitro* studies. In addition, potential association between the expression of GTF2IRD1 and immune cells infiltration levels in PC was explored using ESTIMATE algorithm and single-sample Gene Set Enrichment Analysis (ssGSEA).

## Materials and methods

### Data acquisition

The RNA-sequencing recorded based on FPKM and corresponding clinical data of PC were downloaded from The Cancer Genome Atlas (TCGA, https://cancergenome.nih.gov/) database in March 2020. Of the 177 PC cases obtained, 171 were patients with OS > 1 month. Furthermore, relevant PC datasets were also extracted from the Gene Expression Omnibus (GEO, http://www.ncbi.nlm.nih.gov/geo/) database by using the following search words: ‘pancreatic cancer’ and ‘pancreatic ductal adenocarcinoma.’ The exclusion of ineligible studies was based on the following criteria: (1) studies with fewer than 40 PC samples; (2) studies involving only blood samples, PC cell lines or xenografts. Finally, four GEO datasets, including GSE62452 [platform: GPL6244 (HuGene-1_0-st) Affymetrix Human Gene 1.0 ST Array (transcript (gene) version); 61 non-tumor samples and 69 pancreatic tumor samples], GSE60979 [platform: GPL14550 SurePrint G3 Human GE 8×60K Microarray; 12 non-tumor samples and 49 PC samples], GSE28735 [platform: GPL6244 (HuGene-1_0-st) Affymetrix Human Gene 1.0 ST Array (transcript (gene) version); 45 pairs of pancreatic tumor and adjacent non-tumor tissues], and GSE79668 [platform: GpL11154 Illumina HiSeq 2000 (*Homo sapiens*); 51 pancreatic tumor samples], were used for further study. All datasets (TCGA, GSE62452, GSE60979, GSE28735, and GSE79668) are freely available as public resources. Therefore, local ethics approval was not needed.

### GTF2IRD1 expression analysis

First, the expression level of GTF2IRD1 in PC was analyzed using the Gene Expression Profiling Interactive Analysis (GEPIA; http://gepia.cancerpku.cn/index.html). Then, GSE62452 and GSE60979 datasets were also used to perform differential expression analysis for GTF2IRD1. Moreover, paired differential expression analysis of GTF2IRD1 was also performed based on GSE28735 dataset, which contained the mRNA expression data of 45 pairs of pancreatic tumor and adjacent non-tumor tissues.

### Overall survival analysis

Survival analysis was performed separately to assess the association between GTF2IRD1 expression and overall survival of PC patients in TCGA, GSE62452, and GSE79668 cohorts. Patients in these three PC cohorts were respectively divided into low GTF2IRD1 expression and high GTF2IRD1 expression groups according to the optimal cut-off point obtained from the X-tile 3.6.1 software (Yale University, New Haven, CT, U.S.A.) and KM survival curve was performed using R package survminer. The hazard ratio (HR) with 95% confidence intervals (CI) and log-rank *P*-value were computed [12].

### Correlation analysis of GTF2IRD1 expression and the clinicopathological features

We evaluated the relationship between GTF2IRD1 expression and clinicopathological parameters (such as age, alcoholic history, diabetes status, gender, tumor site, American Joint Committee on Cancer (AJCC) stage, tumor size, recurrence status, histologic grade etc.) through the chi-square test and the Fisher’s exact test (*P*-value <0.05 was considered statically significant).

### Functional enrichment analysis

First, co-expression genes of GTF2IRD1 in the TCGA PC cohort were respectively obtained from the LinkedOmics database (http://www.linkedomics.org). Then, these co-expression genes with the threshold of the Pearson correlated coefficient > 0.55 or < −0.55 and *P*-value <0.05 were enrolled into ConsensuspathDB (http://cpdb.molgen.mpg.de/) and subjected to functional enrichment analysis; *P*-value <0.05 was considered significant [13]. Moreover, Gene Set Variation Analyses (GSVAs), a GSEA method that estimates variation of pathway activity over a sample population, was conducted to calculate the enrichment scores of the potential biological functions of GTF2IRD1 [14]. Then we used Pearson correlation analysis to assess the correlation between GTF2IRD1 expression and these potential biological functions.

### Cell culture and transfection

Human PC cell lines, PANC-1 and Aspc-1 were purchased from the Cell Bank of the Chinese Academy of Science (Shanghai, China). Cells were cultured in RPMI-1640 medium (Invitrogen, Carlsbad, CA, U.S.A.) containing 10% fetal bovine serum (FBS; HyClone, Logan, UT, U.S.A.) and 1% penicillin/streptomycin in a humidified 5% CO_2_ incubator at 37°C. Prior to transfection, cells were seeded on to six-well plates with a density of 1 × 10^6^ cells per well. At 80% confluence, small interfering RNA (siRNA) targeting GTF2IRD1 (sense CAUCGUCCAUGACAAGUCATT and antisense UGACUUGUCAUGGACGAUGGA) and negative control siRNA (NC) were transfected into the cells using Lipofectamine 2000 (Invitrogen). Then, 48 h post transfection, the cells were harvested for further analyses. The efficacy of si-GTF2IRD1 for each cell line was assessed using qRT-PCR.

### MTT assay

PC cell proliferation was measured using 3-(4,5-dimethylthiazal-2-yl)-2,5-diphenyl-tetrazolium bromide (MTT) assay. Cells were cultured in 96-well plates at 2000 cells/well. At the indicated time points, 20 μl of MTT (5 mg/ml; Sigma–Aldrich, St. Louis, MO, U.S.A.) solution was added to each well and the plate was further incubated for 4 h. Culture supernatant was discarded and 100 μl DMSO (Corning Inc., Corning, NY, U.S.A.) was added to each well. Optical density (OD) was determined by measuring absorbance at 490 nm on a microplate reader (Bio-Tek Company, Winooski, VT, U.S.A.).

### Colony formation assay

Cells were plated in six-well plates (1000 cells/well), cultured for 14 days, and stained with 0.1% Crystal Violet. Colony forming units were counted under a microscope.

### Transwell invasion assays

Cells and invasion were examined using Transwell Permeable Supports (Corning Inc., Corning, NY, U.S.A.). Briefly, the transfected cells were allowed to grow to confluence. Cells (10^5^) were suspended in 100 μl serum-free medium and plated in triplicates on to each 8-μm Transwell filter insert of 24-well plates coated with Matrigel (10 mg/l, BD Biosciences, San Jose, CA, U.S.A.). The lower chambers contained 10% FBS. After 18 h, the cells in the upper chamber were removed with a cotton swab. The invasive cells at the bottom of the membrane were fixed with methanol and stained with 0.5% Crystal Violet for 20 min. The stained invaded cells were photographed under an inverted light microscope (magnification, ×100; Olympus Corp., Tokyo, Japan) and nine visual fields were observed in each group, respectively.

### Correlation analysis of GTF2IRD1 and tumor-infiltrating lymphocytes

Using R package estimate, ESTIMATE algorithm was conducted to assess the level of immune infiltration and tumor purity in the TCGA PC, GSE62452, GSE79668, and GSE60979 cohorts, which was based on ssGSEA and generates tumor purity score and immune score [15]. A higher immune score indicates a higher level of the infiltration of immune cells in tumor tissues. PC samples with high tumor purity score show low immune infiltration within tumors. Next, using R package GSVA, ssGSEA was conducted to quantify the activity or enrichment levels of immune cells, functions, or pathways in the TCGA PC, GSE62452, GSE79668, and GSE60979 cohorts. The following four tumor-infiltrating lymphocyte (TIL)-related terms were obtained: CD8^+^ T cells, cytolytic activity, T-cell co-stimulation, and TILs [16]. Correlation analysis between GTF2IRD1 expression and the immune infiltration in PC was performed with Pearson correlation coefficients. The correlation between GTF2IRD1 expression and CD8^+^ T cells was also analyzed using the TIMER web tool (https://cistrome.shinyapps.io/timer/). Furthermore, to further confirm the association between GTF2IRD1 and immune infiltration in PC, we conducted correlation analysis between GTF2IRD1 and the marker genes from TILs using the GEPIA database.

## Results

### GTF2IRD1 overexpression predicts worse prognosis in PC

**Figure 1:**
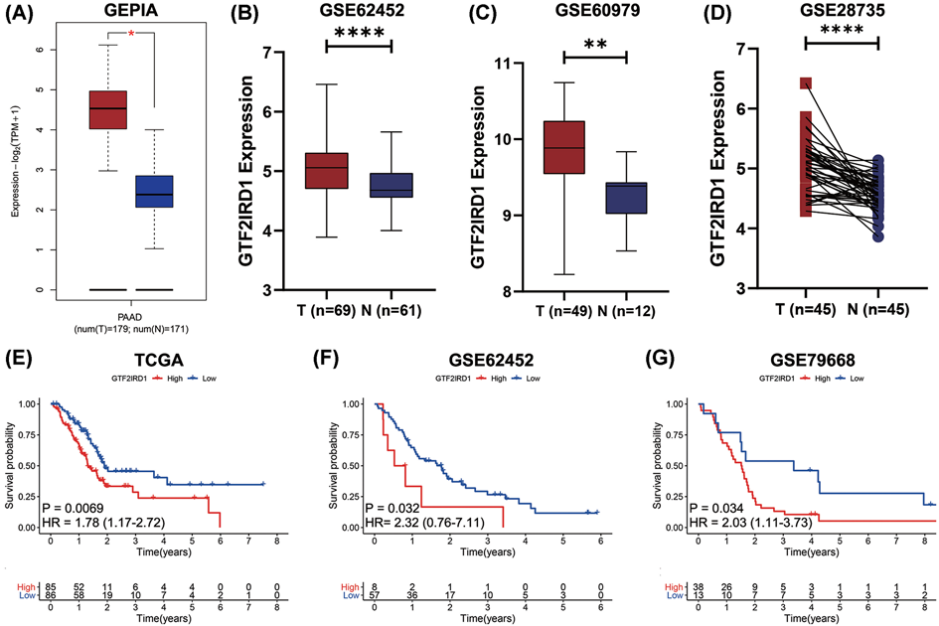
GTF2IRD1 overexpression predicts worse prognosis in PC (**A**–**D**) Multiple databases demonstrated that GTF2IRD1 was overexpressed in PC. (**E**–**G**) KM survival analysis revealed that patients with higher expression of GTF2IRD1 had a shorter OS than those with lower expression of GTF2IRD1 (*P-value <0.05; **P-value <0.01; ****P-value <0.0001).

First, the GEPIA database demonstrated that the expression of GTF2IRD1 was significantly higher in PC tissues than that in normal pancreatic tissues (*P*-value <0.05) (Figure 1A). Moreover, both the GSE62452 and the GSE60979 datasets revealed that GTF2IRD1 was up-regulated in PC tissues compared with normal pancreatic tissues (*P*-value <0.0001) (Figure 1B,C). In addition, paired differential expression analysis based on GSE28735 dataset demonstrated the expression GTF2IRD1 was up-regulated in PC tissues compared with that in the adjacent non-tumor tissues (*P*-value <0.0001) (Figure 1D). Furthermore, we found that GTF2IRD1 was mainly expressed in the cytoplasm of PC cells (Supplementary Figure S1). Importantly, KM survival analysis based on the TCGA PC cohort, the GSE62452 cohort, and the GSE79668 cohort demonstrated that patients with higher expression of GTF2IRD1 had a shorter OS than those with lower expression of GTF2IRD1 (*P*-value <0.05) (Figure 1E–G). Taken together, these data suggested that GTF2IRD1 overexpression may play an unfavorable role in the prognostic prediction of PC.

### GTF2IRD1 overexpression was associated with advanced histologic grade and AJCC stage

The association between GTF2IRD1 expression and clinicopathological features of PC was shown in Table 1. High GTF2IRD1 expression was significantly associated with higher histologic grade (*P*-value = 0.031) and higher AJCC stage (*P*-value = 0.019). These findings indicated that GTF2IRD1 overexpression was involved in the progression of PC.

**Table 1:** Correlation between GTF2IRD1 and clinicopathological features in PC

Parameters	GTF2IRD1 expression		*P*-value
	Low (*n*=91)	High (*n*=86)	
**Age**			
≤60	31 (34.1%)	27 (31.4%)	0.750
>60	60 (65.9%)	59 (68.6%)	
**Gender**			
Female	45 (49.5%)	35 (40.7%)	0.291
Male	46 (50.5%)	51 (59.3%)	
**AJCC stage**			
I	14 (15.4%)	7 (8.1%)	**0.019**
II	68 (74.7%)	78 (90.7%)	
III	3 (3.3%)	0	
IV	4 (4.4%)	0	
Unknown	2 (2.2%)	1 (1.2%)	
**Histologic grade**			
Grade 1	21 (23.1%)	10 (11.6%)	**0.031**
Grade 2	48 (52.7%)	46 (53.5%)	
Grade 3	18 (19.8%)	30 (34.9%)	
Grade 4	2 (2.2%)	0	
Unknown	2 (2.2%)	0	
**Recurrence**			
No	39 (42.9%)	32 (37.2%)	0.444
Yes	52 (57.1%)	54 (62.8%)	
**Alcohol history**			
No	36 (39.6%)	28 (32.6%)	0.343
Yes	49 (53.8%)	52 (60.5%)	
Unknown	6 (6.6%)	6 (6.9%)	
**Diabetes history**			
No	46 (50.5%)	62 (72.1%)	0.131
Yes	22 (24.2%)	16 (18.6%)	
Unknown	23 (25.3%)	8 (9.3%)	
**Tumor size**			
<4	56 (61.5%)	39 (45.3%)	0.058
4	30 (33.0%)	39 (45.3%)	
Unknown	5 (5.5%)	8 (9.3%)	
**Tumor site**			
Head	71 (78.0%)	67 (77.9%)	0.535
Body and tail	12 (13.2%)	16 (18.6%)	
Unknown	8 (8.8%)	3 (3.5%)	

### GTF2IRD1 may down-regulate the expressions of SMAD5 and BMPR1B in PC

SMAD5, BMPR1B, and TGFβR2 were shown to be target genes of GTF2IRD1 in mice [17–19]. Indeed, GTF2IRD1 led to increased expression of TGFβR2, but decreased expression of SMAD5 and BMPR1B [11,18]. Using the GEPIA database, correlation analysis demonstrated that the expressions of SMAD5 and BMPR1B were negatively correlated with the expression of GTF2IRD1 in PC (Supplementary Figure S2). However, no correlation was found between expression of GTF2IRD1 and TGFβR2 in PC. Taken together, GTF2IRD1 may down-regulate the expression of SMAD5 and BMPR1B in PC.

### Functional enrichment analysis of GTF2IRD1 in PC

**Figure 2:**
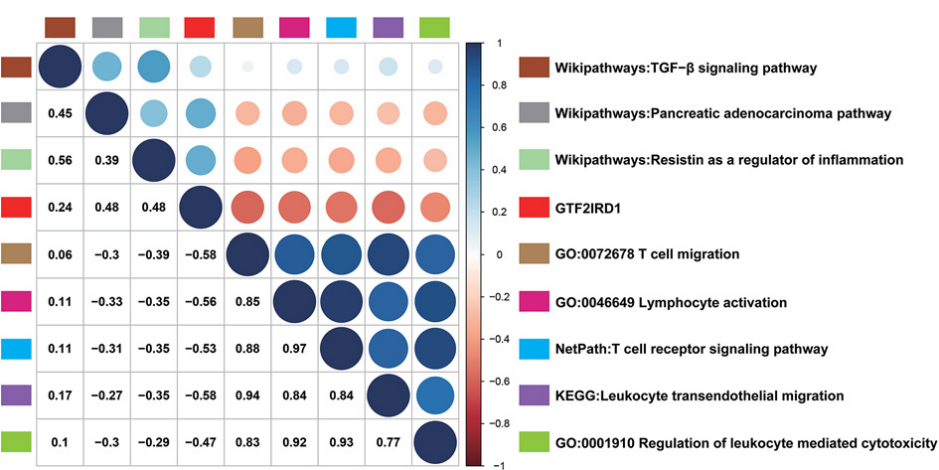
Correlation analysis between GTF2IRD1 expression and function enrichment analysis

We further explored the activities of GTF2IRD1 by analyzing its possible contributions to potential biological pathways in PC. We performed co-expression analysis (Pearson correlated coefficient > 0.55 or < −0.55, *P*-value <0.05) for GTF2IRD1 based on the TCGA cohort, and 367 co-expression genes in the TCGA cohort were enrolled into ConsensuspathDB (http://cpdb.molgen.mpg.de/) and subjected to functional enrichment analysis (*P*-value <0.05) (Tables 2 and 3). GO enrichment analysis revealed that GTF2IRD1 may play an important role in lymphocyte activation, T-cell migration, and regulation of leukocyte-mediated cytotoxicity (Table 2). And GTF2IRD1 expression was negatively correlated with these three biological functions (Cor = −0.56, *P*<0.05 for lymphocyte activation; Cor = −0.58, *P*<0.05 for T-cell migration; Cor = −0.47, *P*<0.05 for regulation of leukocyte-mediated cytotoxicity) (Figure 2). Furthermore, pathway enrichment analysis showed that GTF2IRD1 may be involved in pancreatic adenocarcinoma pathway, TGF-β signaling pathway, T-cell receptor signaling pathway, leukocyte transendothelial migration, resistin as a regulator of inflammation (Table 3). And GTF2IRD1 expression was positively correlated with pancreatic adenocarcinoma pathway (Cor = 0.48, *P*<0.05), TGF-β signaling pathway (Cor = 0.24, *P*<0.05), and resistin as a regulator of inflammation (Cor = 0.48, *P*<0.05), while negatively correlated with T-cell receptor signaling pathway (Cor = −0.53, *P*<0.05), and leukocyte transendothelial migration (Cor = −0.58, *P*<0.05) (Figure 2). These results suggested that GTF2IRD1 overexpression provided necessary support for tumorigenesis, invasion, and the regulation of TILs infiltration in PC.

**Table 2:** GO enrichment analysis of GTF2IRD1 in the TCGA PC cohort

Term	*P*-value	Related genes
GO:0046649 Lymphocyte activation	<0.001	*DOCK8; CD40LG; SLA2; ITGA4; CD209; CCR2; THEMIS; IKZF1; DOCK11; PREX1; PTGER4; FYN; GRAP2; IGF1; RAC1; RHOH; TNFSF8; ITK; CD151; CD5; MEF2C; ITM2A; PTPRC; PPP3CB; CD3G; BCL2*
GO:0072678 T-cell migration	<0.001	*DOCK8; S1PR1; ITGA4; CCR2; CD200R1; CXCL12*
GO:0001910 Regulation of leukocyte-mediated cytotoxicity	0.046	*CD226; PPP3CB; DNASE1L3; PTPRC*

**Table 3:** Pathway enrichment analysis of GTF2IRD1 in the TCGA PC cohort

Term	*P*-value	Related genes
NetPath: T-cell receptor signaling pathway	<0.0001	*ITK; ARHGEF6; SLA2; FYN; HGS; OGN; CD5; ELMO1; GRAP2; PTPRC; GIT2; LCP2; RELA; ITPR1; PPP3CB; CD3G*
Wikipathways: Pancreatic adenocarcinoma pathway	0.0062	*BCL2L1; RAC1; ARHGEF6; RIPK4; PAK4; RELA*
KEGG: Leukocyte transendothelial migration	0.0011	*ITK; RAC1; CDH5; ITGA4; RAPGEF4; RHOH; CXCL12; CLDN4*
Wikipathways: Resistin as a regulator of inflammation	0.0031	*RELA; PLCB3; PLCD3; ITPR1*
Wikipathways: TGF-β signaling pathway	0.036	*SMURF1; MEF2C; ITGB4; RAC1; HGS; PIAS2*

### GTF2IRD1 knockdown suppresses the proliferation and the invasion of PC cells

**Figure 3:**
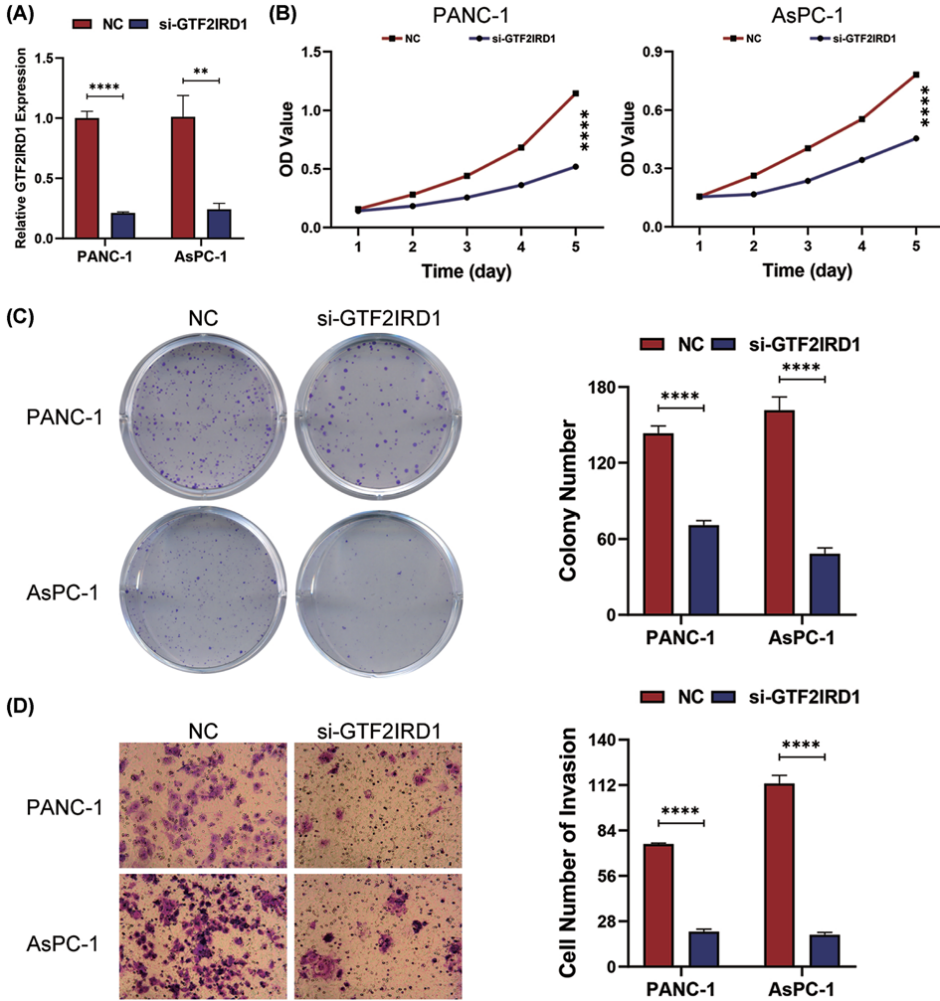
GTF2IRD1 knockdown suppresses the proliferation and invasion of PC cells lines (**A**) RT-PCR analysis validated the knockdown of GTF2IRD1 in PANC-1 and AsPC-1 cells transfected with negative control siRNA (NC) or si-GTF2IRD1. (**B**) Assessment of cell proliferation using the MTT assay. (**C**) Colony formation assay was used to determine the colony-formation ability of PANC-1 and AsPC-1 cells transfected with si-GTF2IRD1. (**D**) Transwell assay was performed to determine the invasive capacity of PANC-1 and AsPC-1 cells transfected with si-GTF2IRD1 (**P-value < 0.01; ****P-value < 0.0001).

To further evaluate the functional role of GTF2IRD1 in tumor cells proliferation and invasion, we transfected PANC-1 and AsPC-1 cells with si-GTF2IRD1. Abrogated levels of GTF2IRD1 in these two cells were validated using RT-qPCR analysis (*P*<0.001) (Figure 3A). Using the MTT assay, we found that cell proliferation was significantly inhibited in GTF2IRD1-depleted PANC-1 and AsPC-1 cells (*P*<0.0001) (Figure 3B). In the plate colony formation assay, the number of colonies in the si-GTF2IRD1 group was significantly lower than that in the NC (control) group (*P*<0.0001) (Figure 3C). Moreover, the transwell assay performed to determine the invasive capacity of PANC-1 and AsPC-1 cells transfected with si-GTF2IRD1 revealed that the number of invasion cells was significantly less in the si-GTF2IRD1 group than that in the NC group (*P*<0.0001) (Figure 3D). These findings suggest GTF2IRD1 is necessary for PC cell proliferation and invasion.

### GTF2IRD1 overexpression correlates with less CD8^+^ T-cells infiltration in PC

**Figure 4:**
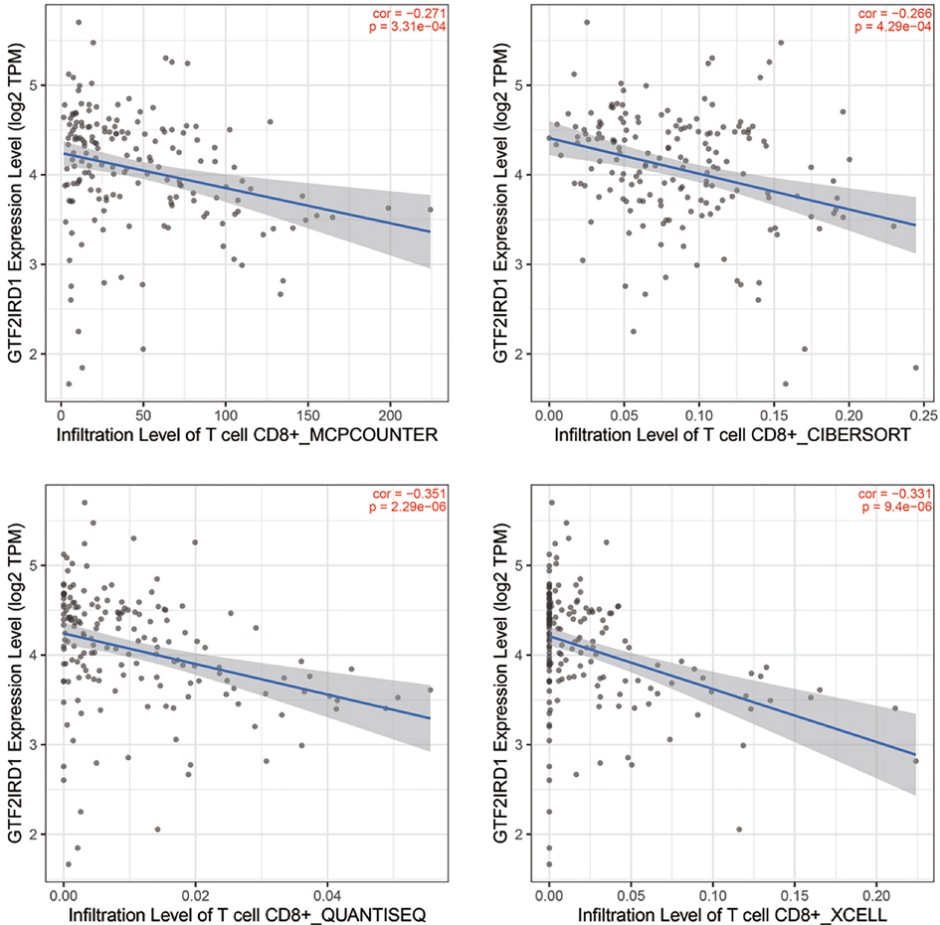
Correlation analysis of GTF2IRD1 expression and CD8^+^ T-cells infiltration in PC using the TIMER web tool

ESTIMATE algorithm demonstrated that GTF2IRD1 expression positively correlated with tumor purity (Cor = 0.40, *P*<0.0001), while negative correlated with immune score (Cor = −0.36, *P*<0.0001) in the TCGA PC cohort (Table 4). Similar results were also observed in GSE62452 cohort, GSE79668 cohort, and GSE60979 cohort (Table 4). Results of ssGSEA were also summarized in Table 4, which demonstrated that GTF2IRD1 expression was negatively associated with the infiltration levels of CD8^+^ T cells, TILs cytolytic activity, and T-cell co-stimulation. Similar results were also observed in the TIMER web tool, in which four different algorithms demonstrated that GTF2IRD1 expression was significantly associated with the infiltration level of CD8^+^ T cells (Cor = −0.271, *P*<0.001 for MCPCOUNTER; Cor = −0.266, *P*<0.001 for CIBERSORT; Cor = −0.351, *P*<0.0001 for QUANTISEQ; Cor = −0.331, *P*<0.0001 for xCell) (Figure 4). Furthermore, we conducted correlation analysis between GTF2IRD1 and the marker genes from TILs using the GEPIA database, which demonstrated GTF2IRD1 overexpression significantly correlated with low expression of these gene markers (e.g. CD8A, CD3D, and CD3E) in PC, but not significant in normal pancreatic tissues (Table 5). These findings strongly suggest that GTF2IRD1 expression negatively correlates with the infiltration and anti-tumor activity of TILs, especially for CD8^+^ T cells.

**Table 4:** Correlation between GTF2IRD1 and immune-related terms based on ESTIMATE algorithm and ssGSEA

Algorithm	Terms	TCGA		GSE62452		GSE79668		GSE60979	
		Cor	*P*-value	Cor	*P*-value	Cor	*P*-value	Cor	*P*-value
ESTIMATE	Tumor purity	0.40	**<0.0001**	0.46	**<0.0001**	0.091	0.53	0.39	**0.0052**
	Immune score	−0.36	**<0.0001**	−0.51	**<0.0001**	−0.21	0.13	−0.44	**0.0016**
ssGSEA	CD8^+^ T cells	−0.41	**<0.0001**	−0.42	**<0.001**	−0.30	**0.031**	−0.41	**0.0037**
	TILs	−0.41	**<0.0001**	−0.51	**<0.0001**	−0.13	0.35	−0.45	**0.0011**
	Cytolytic activity	−0.42	**<0.0001**	−0.44	**<0.001**	−0.26	0.060	−0.34	**0.017**
	T-cell co-stimulation	−0.28	**<0.0001**	−0.48	**<0.0001**	−0.32	**0.020**	−0.44	**0.0014**

P-value < 0.05 was considered statistically significant.

**Table 5:** Correlation between GTF2IRD1 and marker genes of TILs in GEPIA

Gene markers	Tumor		Normal	
	Cor	*P*-value	Cor	*P*-value
CD8A	−0.36	**<0.0001**	0.096	0.21
CD8B	−0.31	**<0.0001**	0.2	**0.0089**
CD3D	−0.23	**0.0017**	0.12	0.12
CD3E	−0.30	**<0.0001**	0.12	0.11

P-value < 0.05 was considered statistically significant.

## Discussion

In the current study, we explored the role of GTF2IRD1 during pancreatic carcinogenesis and its correlation with immune cells infiltration in PC. To the best of our knowledge, this is the first study to prove that GTF2IRD1 might act as a novel oncogene in PC.

Our study demonstrated that GTF2IRD1 was overexpressed in PC, and was positively associated with histologic grade, AJCC stage, and poor survival of PC patients. And previous study by Huo et al. reported that GTF2IRD1 overexpression correlates with poor survival in patients with breast cancer, lung cancer, and ovarian cancer [11]. Additional study by Nambara et al. also identified GTF2IRD1 as an oncogene and unfavorable prognostic factor in colorectal cancer [8]. These findings strongly suggest that GTF2IRD1 is a potential oncogene and unfavorable prognostic biomarker in human cancers, including PC.

Next, we assessed the underlying mechanisms through which GTF2IRD1 promoted PC progression. Through pathway function enrichment analysis, we found that GTF2IRD1 expression was positively correlated with pancreatic adenocarcinoma pathway and TGF-β signaling pathway. Our schema was further supported by the fact that GTF2IRD1 knockdown significantly inhibited cell proliferation and invasion in PC cells. Thus, we proposed that GTF2IRD1 overexpression promoted tumor progression in PC. As a transcriptional factor, GTF2IRD1 was shown to induce transcriptionally repression of SMAD5 and BMPR1B [11,18]. Consistently, we observed negatively correlation between GTF2IRD1 and SMAD5, also between GTF2IRD1 and BMPR1B, suggesting GTF2IRD1 may down-regulate the expression of SMAD5 and BMPR1B in PC. Previous studies reported that SMAD5 was a tumor suppressor, which served as a crucial intracellular inhibitor of TGF-β signaling pathway [20]. Similarly, BMPR1B was also reported to be a transmembrane receptor mediating TGF-β signaling pathway [21–23]. Inhibition of BMPR1B was found to enhance TGF-β signaling [23]. Thus, overexpression of GTF2IRD1 may down-regulate the expression SMAD5 and BMPR1B, thereby activating TGF-β signaling pathway. These results suggested that GTF2IRD1 overexpression might promote tumor progression through activating TGF-β signaling pathway in PC. However, early study by Hasegawa et al. suggested that GTF2IRD1 represses adipose tissue fibrosis through inhibiting TGF-β signaling pathway [24]. Besides, Nambara et al. reported that GTF2IRD1 overexpression might result in inhibition of TGF-β signaling pathway in colorectal cancer [8]. These results suggest the tissue specificity of GTF2IRD1 in regulating TGF-β signaling pathway. Further experimental studies are needed to assess whether GTF2IRD1 play a vital role in the progression of PC by regulating TGF-β signaling pathway.

To note, pathway enrichment analysis demonstrated that GTF2IRD1 expression was positively correlated with resistin as a regulator of inflammation and was negatively correlated with T-cell receptor signaling pathway, and leukocyte transendothelial migration. Moreover, GO enrichment analysis demonstrated that GTF2IRD1 expression was negatively correlated with lymphocyte activation, T-cell migration, and regulation of leukocyte-mediated cytotoxicity. Therefore, we hypothesized that GTF2IRD1 expression could be a potential marker for decreased TIL infiltration in the tumor immune microenvironment. Consistently, ESTIMATE algorithm also demonstrated that high expression of GTF2IRD1 was correlated with high tumor purity and low immune score, indicating that GTF2IRD1 overexpression may induced immune suppression in PC. And ssGSEA revealed that GTF2IRD1 overexpression was significantly correlated with low infiltration of TILs, especially for CD8^+^ T cells. Besides, GTF2IRD1 overexpression was correlated with lower cytolytic activity and T-cell co-stimulation. Similar results were also observed in the TIMER web tool. All these findings supported the role of GTF2IRD1 overexpression in suppressing the infiltration and anti-tumor effect of CD8^+^ T cells, although the underlying mechanism needs to be further studied.

Increasing evidences suggested that the excessive secretion of TGF-β in TME was closely related to impaired tumor-killing effect of CD8^+^ T cells [25,26]. And it has also been reviewed that blocking TGF-β signaling pathway could improve the efficacy of immune checkpoint inhibitors [27]. Besides, TGF-β also promoted EMT, which led to anti-tumor immune suppression and ineffective treatment of immune checkpoint inhibitors [28–32]. In our study, function enrichment analysis revealed that GTF2IRD1 expression positively correlated with TGF-β signaling pathway, indicating the potential role of GTF2IRD1 in the activation of TGF-β signaling pathway. Thus, we proposed that blocking TGF-β by targeting GTF2IRD1 might be an effective therapeutic strategy that is complementary to immune checkpoint inhibitors. Future studies should be conducted to figure out how GTF2IRD1 regulate CD8^+^ T-cell infiltration through TGF-β signaling pathway in PC.

Our study provides new insights into the tumor immune microenvironment and immune-based therapies for PC. To the best of our knowledge, the current study is the first to provide clear evidence for the association between GTF2IRD1 expression and CD8^+^ T cells infiltration in PC. However, some limitations exist in the present study. First, this is a retrospective study based on publicly available datasets. Therefore, the quality of data can influence the study outcomes. Second, the potential role of GTF2IRD1 in pancreatic carcinogenesis was validated *in vitro*, but not *in vivo*. Third, further experimental studies should be performed to validate the potential role of GTF2IRD1 in regulating immunosuppression in the TME in future. Moreover, the hypothesis about the potential role of GTF2IRD1 in TGF-β signaling pathway remains to be studied in the future studies.

## Conclusions

In summary, our study identified GTF2IRD1 as an unfavorable prognostic biomarker. And GTF2IRD1 overexpression promotes tumor progression and correlates with less CD8^+^ T-cells infiltration, which might be a novel therapeutic target in PC.

## Supplementary Material

Supplementary Figures S1 and S2

## Data Availability

All data used and analyzed in the present study are available from the correspondence upon reasonable request.

## Abbreviations

AJCC: American Joint Committee on Cancer
FBS: fetal bovine serum
FPKM: fragments per kilobase per million
GEO: Gene Expression Omnibus
GEPIA: Gene Expression Profiling Interactive Analysis
GSVA: Gene Set Variation Analysis
GTF2IRD1: general transcription factor II-I repeat domain-containing protein 1
KM: Kaplan-Meier
MTT: 3-(4,5-dimethylthiazal-2-yl)-2,5-diphenyl-tetrazolium bromide
OS: overall survival
PC: pancreatic cancer
siRNA: small interfering RNA
ssGSEA: single-sample Gene Set Enrichment Analysis
TCGA: The Cancer Genome Atlas
TIL: tumor-infiltrating lymphocyte
TME: tumor microenvironment
