# Supplementary material for: GTF2IRD1 overexpression promotes tumor progression and correlates with less CD8+ T cells infiltration in pancreatic cancer
Source: Biosci Rep. 2020 Sep 28;40(9):BSR20202150. doi: 10.1042/BSR20202150 (PMC7527428; doi:10.1042/BSR20202150)
Supplement: Supplementary Figures S1 and S2 [file BSR-2020-2150_supp.pdf]

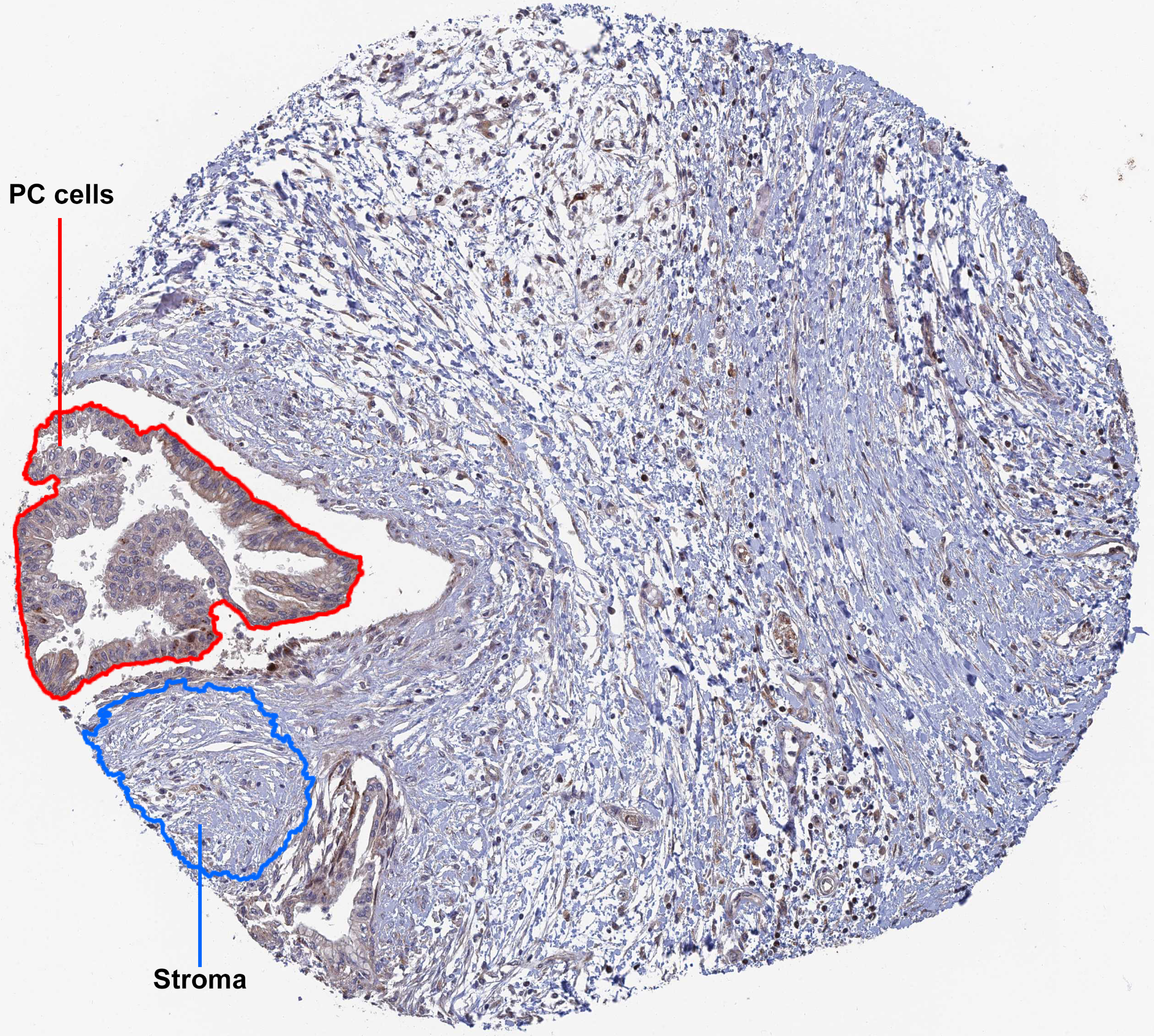

PC cells
Stroma

Figure S1. The Human Protein Atlas database demonstrated GTF2IRD1 was mainly expressed in the cytoplasm of PC cells, while slightly expressed in the stromal cells.

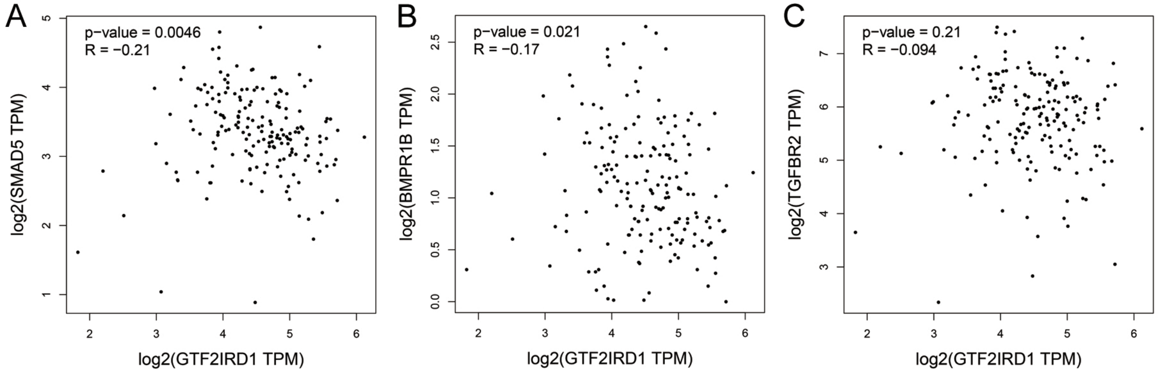
A
p−value = 0.0046
R = −0.21
log2(SMAD5 TPM)
log2(GTF2IRD1 TPM)
B
p−value = 0.021
R = −0.17
log2(BMPR1B TPM)
log2(GTF2IRD1 TPM)
C
p−value = 0.21
R = −0.094
log2(TGFBR2 TPM)
log2(GTF2IRD1 TPM)

Figure S2. Correlation analysis of GTF2IRD1 and its known target genes. (A-B) The expressions of SMAD5 and BMPR1B were negatively correlated with the expression of GTF2IRD1 in PC. (C) The expression of TGFβR2 was not significantly correlated with the expression of GTF2IRD1 in PC.
